# The effects of preferential A- and C-fibre blocks and T-type calcium channel antagonist on detection of low-force monofilaments in healthy human participants

**DOI:** 10.1186/s12868-015-0190-2

**Published:** 2015-08-13

**Authors:** Saad S. Nagi, James S. Dunn, Ingvars Birznieks, Richard M. Vickery, David A. Mahns

**Affiliations:** School of Medicine, University of Western Sydney, Locked Bag 1797, Penrith, NSW 2751 Australia; Neuroscience Research Australia, PO Box 1165, Randwick, NSW 2031 Australia; School of Medical Sciences, UNSW Australia, Sydney, NSW 2052 Australia

**Keywords:** C-tactile fibre, Glabrous skin, Tactile detection, Calcium channel Cav3.2, von Frey filament

## Abstract

**Background:**

A myriad of studies have argued that tactile sensibility is underpinned exclusively by large myelinated mechanoreceptors. However, the functional significance of their slow-conducting counterparts, termed C-low threshold mechanoreceptors (C-LTMRs), remains largely unexplored. We recently showed the emergence of brush- and vibration-evoked allodynia in human hairy and glabrous skin during background muscle pain. The allodynia persisted following the preferential blockade of myelinated fibres but was abolished by the preferential blockade of cutaneous C fibres, thereby suggesting a pathway involving hairy skin C-LTMRs and their functional counterparts in glabrous skin in this phenomenon. In the present study, we tested the effects of preferential A- and C-fibre conduction blocks and pharmacological blockade of T-type calcium channel Cav3.2 (expressed selectively on small-fibre LTMRs) on monofilament detection thresholds in healthy participants by compression, low-dose intradermal anaesthesia (xylocaine 0.25 %) and selective T-channel antagonist, TTA-A2.

**Results:**

We found that all participants could detect monofilament contacts (as low as 1.6 mN) within the innocuous tactile range regardless of the preferential blockade of myelinated fibres. Furthermore, during the compression block no subject reported a switch in modality from touch to pain. That is, the low-force monofilament contacts were always perceived as non-painful. However, there was a small but significant elevation of monofilament thresholds (~2 mN) in the glabrous skin following the compression block. Importantly, no differences were found in the thresholds across hairy and glabrous regions while the myelinated fibres were conducting or not. The preferential blockade of C fibres in the glabrous skin (with myelinated fibres intact) also resulted in a small but significant elevation of tactile thresholds. Furthermore, the use of T-channel blocker in the glabrous skin during compression block of myelinated fibres resulted in complete abolition of monofilament sensibility within the innocuous tactile range (tested up to ~20 mN).

**Conclusions:**

These observations suggest that C-LTMRs need not be regarded as a redundant tactile system, but appear to complement normal large-myelinated-fibre tactile function. Convergent findings in glabrous and hairy skin lend support for an underlying system of innocuous mechanoreception with Cav3.2-expressing unmyelinated fibres.

## Background

It is widely appreciated that large myelinated mechanoreceptors are the underlying neural substrate of sensory-discriminative touch, which includes the perception of pressure, vibration/texture, skin stretch, and in case of hairy skin, displacement of hair follicles [1–4]. Conversely, little is known about the contribution of their unmyelinated counterparts, termed C-low threshold mechanoreceptors (C-LTMRs), to the tactile domain (N.B. in this paper, the abbreviation ‘C-LTMRs’ refers to C-low threshold mechanoreceptors across all species, including the so-called C-tactile fibres in humans). Based on a phylogenetic classification, C mechanoreceptors represent a very ancient system of slowly conducting unmyelinated afferents. This afferent class was first reported by Zotterman [5] in the hairy skin of cat. Over the years, the existence of C-LTMRs, although once deemed an evolutionary vestige [6], has been confirmed in the hairy skin of multiple species including toad, mouse, rat, rabbit and monkey [6–10]. In addition, despite initial failure to discover this afferent class in humans, subsequent microneurography studies have confirmed their ubiquitous distribution in human hairy skin [11–15].

There is no clear evidence yet for the existence of C-LTMRs in the glabrous skin. In saying that, we recently provided psychophysical evidence for the existence of an apparent ‘*functional homologue*’ of C-LTMRs in human glabrous skin based on: (1) the production of cutaneous allodynia during background muscle pain by applying focal vibratory stimuli and gentle brushing to glabrous (and hairy) skin at stroking speeds deemed optimal for C-LTMR activation in hairy skin; (2) the persistence of allodynia following the preferential blockade of myelinated fibres by compression; (3) the abolition of allodynia following the preferential blockade of C fibres by injecting a small amount of low-dose anaesthetic into the glabrous skin [16]. Consistent with that, it has been reported that vesicular glutamate transporter 3 (VGLUT3), a marker for C-LTMRs, is found in a subset of dorsal root ganglia (DRG) neurons that innervate the glabrous skin [17]. Furthermore, the allodynia evoked by mechanical stimulation of the glabrous skin was diminished in the VGLUT3-knockout mice, consistent with our observations of C-LTMR-mediated allodynia in human glabrous skin [16, 17]. However, depending on the type of molecular/genetic markers used and the degree of selectivity of their expression within DRG neurons, other studies have argued against the existence of a phenotypically identical class of hairy skin C-LTMRs in the glabrous skin [8, 18]. Recently, T-type calcium channel Cav3.2 has emerged as a selective marker for the small-fibre LTMRs expressing VGLUT3 and other known markers for C-LTMRs including tyrosine hydroxylase (TH) and chemokine-like protein TAFA4 [19]. This calcium channel appears to regulate the perception of light touch (using ~6-mN monofilament stimulation) and allodynia given the diminution of these phenomena in the C-LTMR specific knockout mice [19]. Indeed, we have recently shown the abolition of experimentally evoked allodynia in healthy human participants by localised blockade of T-type calcium channels [20].

In the current study, we measured monofilament perceptual thresholds in both hairy and glabrous skin of healthy participants with a full complement of sensory fibres. These measurements were carried out in the normal state as well as following preferential blocks of myelinated and unmyelinated fibres. The effects of nerve conduction blocks on mechanical contact sensibility are important in the light of (1) somewhat preserved tactile sense in the hairy skin of two large-fibre deafferented patients with detection thresholds of 23 and 46 mN and (2) our recent observation of tactile allodynia in the glabrous skin which was preserved following the blockade of myelinated fibres but was abolished by the blockade of C fibres [16, 21]. Indeed, if the hairy skin C-LTMR or its functional homologue exists in the glabrous skin, then any effects of myelinated-fibre blockade on tactile thresholds must be comparable across the two skin types. Consistent with that, we found no significant differences in the tactile thresholds across the hairy and glabrous test sites while the myelinated fibres were conducting or not. Furthermore, we observed a small but significant elevation in tactile thresholds when the C fibres were preferentially blocked in glabrous skin (myelinated fibres intact), suggesting that C fibres contribute to normal tactile activity. Importantly, we found that the localised blockade of Cav3.2 channels in the glabrous skin during compression block of myelinated fibres resulted in complete loss of tactile detection within the innocuous tactile range.

## Methods

Twenty-one healthy human subjects (13 males and 8 females) aged 18–40 years with no reported musculoskeletal or neurological disorder were recruited for this study. Informed written consent was obtained from each subject prior to the experiment. The study was approved by the Human Research Ethics Committee (approval number: H9190) of the University of Western Sydney in accordance with the revised Declaration of Helsinki. Subjects sat with their hand resting on a wooden hand-model affixed to the bench top. Before starting the experiment, each subject was asked to trim down the hair on the dorsal aspect of their hand (overlying the fifth metacarpal) in order to prevent any cues associated with movement of hair follicles during the application of test stimuli.

In this study, the *first* aim was to determine whether the effect of myelinated-fibre block on tactile detection is similar across the hairy and glabrous regions. This was tested by employing preferential conduction block of the myelinated fibres by compression of ulnar nerve, and measuring tactile thresholds in the affected hairy and glabrous regions prior to and during the block. The *second* aim was to determine whether the C-LTMR homologue in glabrous skin contributes to normal tactile activity. This was tested by employing preferential conduction block of C fibres by injecting a small amount of low-dose intradermal anaesthetic in the distal palmar pad of little finger, and comparing tactile thresholds between the all-fibre-intact and C-fibre-blocked conditions. The *third* aim was to test whether the residual (C-fibre-mediated) tactile sensibility in glabrous skin following the blockade of myelinated fibres is underpinned by the Cav3.2 calcium channels. This was tested by injecting a small amount of T-type calcium channel antagonist into the distal palmar pad of little finger, and comparing the residual tactile thresholds (myelinated fibres blocked) between the two conditions.

### Monofilament detection thresholds

Tactile detection thresholds were measured using Semmes–Weinstein monofilaments (von Frey filaments) at three different test sites: hairy skin (H) of dorsal hand overlying fifth metacarpal and corresponding glabrous region of palm (P), and glabrous skin of little finger (D5). Five equidistant (10-mm apart) asymmetrical points were marked within each test site. *Low*-*force* monofilaments (0.08–19.6 mN; Bioseb, Chaville, France) were presented five times (in a pseudo-random order) with a hold phase of 2–3 s, and the subjects were verbally prompted to report whether a stimulus was felt or not. In the affirmative, the subjects were asked to report whether the resulting sensation was painful or non-painful. If the subject failed to detect at least four out of five stimulations, then a monofilament with greater force was used. To ensure this was not a product of chance, accurate detection of at least 80 % of stimulations in four out of five repeats of the same (weakest) monofilament was taken as the detection threshold (mN). In order to account for response learning/attentional bias, at least one false prompt was introduced during each set of five stimulations. Subjects were blinded to visual cues for the period of tactile testing.

### Preferential block of A fibres by compression—C fibres intact

A conduction block of ulnar nerve was attempted in all subjects except one who reported insensitivity to heat pain (>50 °C)—a measure of C-fibre function—during control testing. However, an effective blockade could only be achieved in 12 out of 20 subjects as determined by repeated sensory testing with no reports of background or evoked pain (to otherwise non-painful stimuli) for the entire duration of testing while the block was in effect (~30 min). The conduction block was induced by placing a small metal slab just proximal to the medial epicondyle of humerus—as reported previously [16]—whilst the hand was gently resting on a wooden hand-model with the elbow in a semi-flexed position. In order to gauge the progression of the block, vibratory (20 Hz–20 µm; Piezo Tactile Stimulator, Dancer Design, UK) and thermal (brass rods applied at temperatures of ~15 and ~40 °C with a contact time of ~5 s) sensibilities were tested in the skin regions innervated by ulnar nerve. In addition, the same stimuli were applied to the index finger (innervated by median nerve) and the contralateral little finger in order to compare the somatosensory sensibility between the intact and affected regions. In the *simple detection tests*, the subjects were verbally prompted to report whether a stimulus was felt or not. In the affirmative, the subjects were asked to report the quality and perceived area of sensation. The loss of focal vibration and innocuous cold sensation was taken as indication that the large- and small-diameter myelinated fibres were blocked. Likewise, the intactness of C-fibre conduction was determined by a preserved appreciation of the warm/heat sensation [1, 2, 22].

To support the observations from simple detection tests, non-painful cold (Aδ-fibre mediated) and heat-pain (C-fibre mediated) detection thresholds were also measured [20]. These tests were carried out in the glabrous skin of little finger (ulnar territory) with a small 5- × 5-mm thermode (baseline temperature: 32 °C; rate of change: 1 °C/s) attached to a computer-controlled sensory testing device, using the method of limits (Thermal Sensory Analyzer II, Medoc Ltd., Ramat Yishai, Israel). The subject was instructed to press a button as soon as a change of temperature was detected, and at the point when pain was elicited. These measurements were carried out in duplicates, in addition to a practice run prior to the start of actual testing in order to familiarise the subjects with the protocol. The test site was changed, usually to a different phalanx of little finger, following the application of a noxious stimulus in order to avoid any effects of peripheral sensitization on our measurements. The amount of time taken to achieve a compression block varied across subjects, but it always took effect within an hour—consistent with our earlier observations [16].

Following the blockade of myelinated fibres, monofilament detection thresholds for all three test sites (H, P and D5) were measured in seven participants using the aforementioned protocol.

### Preferential block of C fibres by low-dose intradermal anaesthesia—myelinated fibres intact

In a subset of subjects (*n* = 7), additional observations were obtained across two subsequent sessions by injecting a small amount (~0.2 ml) of low-dose anaesthetic (xylocaine 0.25 %) or normal saline (0.9 %) into a ~1-cm region in the distal palmar pad of little finger. Subjects were not informed whether the injection contained anaesthetic or saline. The anaesthetic was injected in order to preferentially block the cutaneous C fibres [1, 2, 16], and thus ascertain whether C-fibre input contributes to punctate tactile detection in glabrous skin. Whereas, saline was injected in order to test whether any effect on tactile detection attributed to anaesthetic was simply a result of changes in local skin mechanics (as a result of trauma/fluid accumulation) or in fact the blockade of C-fibre input from the test site.

Test points were marked at 2-mm intervals on the distal palmar pad of little finger. The effectiveness of C-fibre block in glabrous skin (typically manifested in less than 5 min) was confirmed by the abolition of warm/heat sensation (~40 °C, brass rod), whereas preserved appreciation of vibration (20 Hz–20 µm), cool (~15 °C, brass rod) and pinprick (‘sharp’ pain—prodding with a sterile hypodermic needle or monofilaments in noxious range >100 mN) sensations was taken for the intactness of myelinated-fibre conduction [1, 2, 22]. These tests were carried out at regular intervals—typically once every two sets of monofilament detection trials—in order to confirm that only the C fibres (warm sensibility) were blocked.

### Localised blockade of T-type calcium (Cav3.2) channels in the compression condition

We examined whether the C-fibre contribution from glabrous skin is dependent on the Cav3.2 calcium channels by injecting 0.5 ml of 1 mg/ml TTA-A2—a selective T-type calcium channel antagonist—into the distal palmar pad of little finger in 5 participants. 5-mg TTA-A2 powder was dissolved in 0.5-ml alcohol and then 0.1-ml aliquots were diluted in 0.9-ml normal saline to produce a 1 mg/ml solution. This is consistent with our earlier work where the same dose of TTA-A2 abolished the C-LTMR-mediated allodynia [20]. As Cav3.2 channels are selectively expressed on Aδ- and C-LTMRs [19], we tested the effects of pharmacological T-type channel suppression during the compression block of myelinated fibres (i.e. Aβ and Aδ blocked). Thereafter, we measured the tactile thresholds from the TTA-A2-treated region—distal palmar pad of little finger—as per the aforementioned protocol. In an additional participant, we administered the antagonist in half dose (0.5 ml of 0.5 mg/ml) in order to test whether any dose-dependent effect was observable in the resulting tactile thresholds. In another additional participant, control observations were made where 0.5 ml of the same solution (alcohol diluted in saline) was administered but without the TTA-A2 in order to test whether any changes to tactile detection were simply an effect of alcohol or changes to local skin mechanics, or whether it was attributable to the inhibition of Cav3.2 channels on C-LTMRs.

### Statistical analyses

For tactile thresholds, the fibre values (categories) are given in mN, as medians and quartiles (*Q*: 25th and 75th percentiles). In order to test for significant differences (*P* < 0.05), Friedman test was used for comparisons *across test sites* and Wilcoxon matched-pair signed rank test was used for comparison *across treatments*. Cooling and heat-pain thresholds are given in °C, as means ± standard deviations (SD) of two trials. For significance testing (*P* < 0.05), differences *across treatments* were evaluated using Paired *t* test. All statistical comparisons were performed using GraphPad Prism (v.6.04 for Windows; GraphPad Software, San Diego, CA, USA; RRID:rid_000081).

## Results

In the current study, monofilament detection thresholds were measured prior to and following preferential blocking of A and C fibres in hairy and glabrous skin. At detection threshold, the inclusion of false prompts at regular intervals (one-sixth of trials) evoked a false positive response *once* in two subjects following compression or anaesthetic. One of these subjects also reported two false positives in the control condition.

In all compression experiments, the efficacy of the block was confirmed by the abolition of vibration and cool sensations but intact appreciation of warm/heat sensation. Consistent with blockade of Aδ fibres, threshold testing (°C) revealed obvious differences in the detection thresholds for cooling under the two conditions (*intact*: 23 ± 7; *compression*: 10 ± 9; Paired t test: *P* = 0.0005). Furthermore, when all nerves were intact, the detection of cooling was described as innocuous whereas, following compression, the first detected sensation was of pain (i.e. loss to innocuous cooling = Aδ fibres blocked). Consistent with preserved C-fibre function, there were no differences in the absolute heat pain threshold between the intact (45 ± 2) and compression (45 ± 3) conditions.

### Comparable mechanical thresholds across skin types in intact and compression conditions

**Fig. 1:**
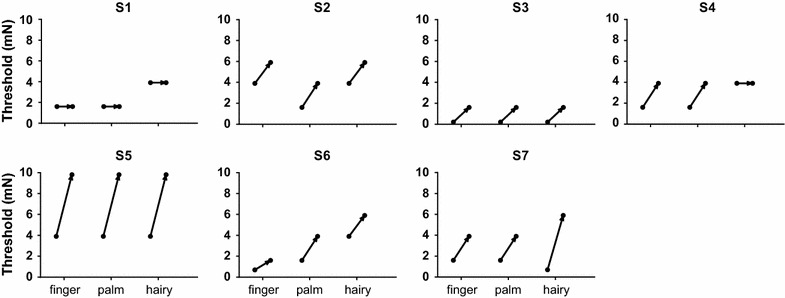
Preserved capacity to detect low-force monofilaments following preferential block of myelinated fibres. Monofilament detection thresholds (mN) were measured in the ulnar territory of the glabrous (*finger* and *palm*) and *hairy* skin of the hand prior to, and following, conduction blockade of myelinated fibres. Threshold data are shown for each subject (*S1*–*S7*) with an *arrow* linking the intact and compression values. In all subjects, the capacity to detect weak monofilament contacts was preserved in hairy and glabrous regions despite the blockade of myelinated fibres. In fact, with the exception of one subject (*S5*), all of them could reliably detect contact forces below 6 mN in both skin types, and in three of them (*S1*, *S3* and *S6*) the post-compression threshold was below 2 mN in the glabrous region

**Fig. 2:**
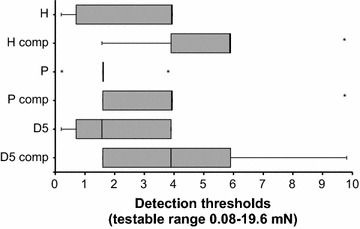
Comparable mechanical thresholds across skin types in intact and compression conditions. Monofilament detection thresholds (mN) were measured in the ulnar territory of the finger (D5), palm (P) and dorsal hand (H) prior to and following compression (comp) of myelinated fibres, and presented as a *box*-and-*whisker plot*. When the intact and compression values for each individual test site were compared, significant differences emerged for the glabrous skin sites, but not for the hairy skin. However, when thresholds were compared between the hairy and glabrous test regions, no significant differences were found irrespective of whether the myelinated fibres were conducting or not. Outliers are marked with an *asterisk* (*) i.e. any data point below Q_1_ − 1.5 × IQR or above Q_3_ + 1.5 × IQR

When all fibres were intact, the median values (mN) for monofilament detection for hairy (H) and glabrous (P and D5) skin sites were 3.9 (*Q* 0.7–3.9), 1.6 (*Q* 1.6–1.6) and 1.6 (*Q* 0.7–3.9), respectively. In this condition, no significant differences were found across the three sites (Friedman test: *P* = 0.46). Following the blockade of myelinated fibres, a change in the quality of touch was reported such that the focal and familiar sense of touch was replaced with a dull and delayed percept that was often perceived as vaguely localised. The post-compression median values for H, P and D5 were found to be 5.9 (*Q* 3.9–5.9), 3.9 (*Q* 1.6–3.9) and 3.9 (*Q* 1.6–5.9), respectively. When the intact and compression values for each individual test site were compared using Wilcoxon matched-pairs signed rank test, significant differences emerged for the glabrous skin sites, palm (*P* = 0.03) and finger (*P* = 0.03), although not for the hairy skin (*P* = 0.06). However, when post-compression threshold values were compared across sites, no significant differences emerged (Friedman test: *P* = 0.07). Furthermore, the capacity to detect low-force monofilament stimuli was preserved in both skin types, and at no point a switch in modality from touch to pain was reported. In fact, with the exception of one subject, all of them could reliably detect tactile forces below ~6 mN regardless of whether the stimulus was applied to hairy or glabrous skin and while the myelinated fibres were conducting or not. Indeed, in three subjects, the post-compression detection threshold in the digital glabrous skin was as low as 1.6 mN. Figures 1 and 2 show the tactile threshold data of all subjects in both intact and compression conditions and across all three test sites.

### Elevated tactile thresholds in glabrous skin following preferential block of C fibres

A preferential block of C-fibre inputs arising from digital glabrous skin was attempted by administering a low-dose intradermal anaesthetic. The preferential effect of the C-fibre block was confirmed by the imperceptiveness to warm/hot stimuli but preserved perception of vibratory, cold and pinprick stimuli. Based on this criterion, a preferential block of C fibres could not be achieved in 1 subject since cold sensibility appeared to be affected as well, and this subject was not included in the following data.

**Fig. 3:**
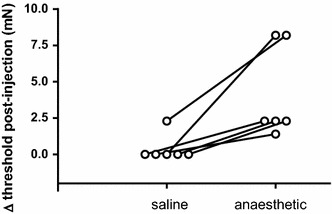
Elevated tactile thresholds following preferential block of C fibres in glabrous skin. Monofilament detection thresholds (mN) were measured in the distal palmar pad of little finger following treatments with *normal saline* and *low*-*dose anaesthetic*. Changes in individual detection thresholds are shown for saline (i.e. saline-control) and anaesthetic (i.e. anaesthetic-control) conditions with a line linking the ∆ saline and ∆ anaesthetic values. In all subjects, treatment with anaesthetic, but not saline, resulted in elevated thresholds for monofilament detection

Prior to anaesthetic and saline treatment, the control median values (mN) for monofilament detection were 1.6 (*Q* 1.3–1.6) for the anaesthetic session and 1.2 (*Q* 0.6–2.2) for the saline session. The monofilament detection thresholds across the two control sessions were found to be statistically indistinguishable, as shown by Wilcoxon matched-pairs signed rank test (*P* = 0.75), which attests to the reproducibility of our testing protocol. Following the blockade of C fibres, the monofilament detection threshold increased to a median value of 3.9 (*Q* 3.3–9.8). The post-anaesthetic detection threshold was found to be significantly different from the control condition (Wilcoxon matched-pairs: *P* = 0.03). In contrast, following saline treatment, the median threshold was found to be 1.2 (*Q* 0.6–3.9), which was identical to the control threshold (Wilcoxon: *P* > 0.99). In terms of the quality of touch, the subjects did not report any obvious differences between the two conditions, that is, when all fibres were intact and following the blockade of C fibres. Figure 3 shows the changes in individual tactile thresholds for saline (i.e. saline-control) and anaesthetic (i.e. anaesthetic-control) conditions.

### Abolition of innocuous tactile detection in glabrous skin following localised Cav3.2 blockade in the compression condition

**Fig. 4:**
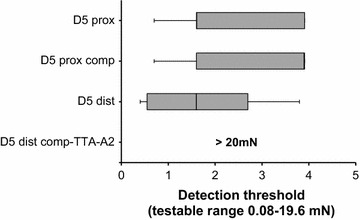
Abolition of post-compression tactile sensibility following Cav3.2 blockade in glabrous skin. Monofilament detection thresholds (mN) were measured in the proximal (prox) and distal (dist) phalanges of the little finger (D5) prior to and following compression (comp) of myelinated fibres and treatment with T-type calcium channel antagonist, TTA-A2. The data are presented as a *box*-and-*whisker plot*. While all participants could detect low-force monofilament contacts in the compression condition, the subsequent blockade of Cav3.2 channels in glabrous skin abolished their capacity to detect monofilament contacts across the innocuous range (up to ~20 mN)

In the intact condition, the median values (mN) for monofilament detection were 1.6 (*Q* 1.6–3.9) and 1.6 (*Q* 0.6–2.8) for the proximal and distal palmar pads of little finger, respectively. Following compression block (*n* = 7), once the monofilament sensibility was found to be intact—tested using an abridged protocol of a single set of five stimulations in the proximal phalanx (median 3.9, *Q* 1.6–3.9)—the distal phalanx was treated with the calcium channel antagonist. Across all participants, the capacity to detect monofilament contacts within our innocuous testable range (up to ~20 mN) was completely lost in the treated region. However, other types of C fibres were still intact as indicated by detection of warm/hot rods (~40 °C) and noxious punctate stimuli (>40 mN, perceived as painful) applied to the treated region. In one participant where the calcium channel antagonist was administered in half dose, the innocuous tactile sensibility was still intact but reduced in sensitivity (*control*: 0.2 mN; *post*-*compression*-*TTA*-*A2*: 5.9 mN). In an additional participant where the same volume of alcohol and saline was administered but without the calcium channel antagonist, only a slight change in tactile threshold was reported between the control condition (0.7 mN) and following compression with saline-alcohol injection (1.6 mN). These observations suggest that the C-fibre contribution to tactile detection is underpinned by Cav3.2 calcium channels—expressed on C-LTMRs—given the complete abolition of subjects’ capacity to detect innocuous monofilament contacts following localised blockade of Cav3.2 channels in the compression condition. Figure 4 shows the tactile threshold data for the intact and compression conditions and the abolition of low-force monofilament detection following blockade of Cav3.2 channels.

## Discussion

In this study, we have shown that the blockade of myelinated afferent fibres need not result in a complete loss of tactile function. Indeed, our subjects could reliably detect low-force punctate mechanical stimuli on both the hairy and glabrous regions of skin with only C fibres intact. Hence, the residual C-fibre-mediated peripheral input could provide a reliable indication of mechanical events on the surface of both skin types. It is noteworthy that tactile sensitivity in the glabrous skin, which is often suggested to be devoid of C-LTMRs, was more or less indistinguishable from the hairy skin. Indeed, this finding serves as further evidence for the existence for an underlying network of C-LTMRs in human glabrous skin that, akin to their hairy skin counterparts, can encode punctate mechanical stimuli. Intriguingly, this role of C-LTMRs appears to be not just limited to experimental conditions of myelinated-fibre blockade but extends to normal conditions with a full complement of sensory fibres. Following the blockade of C fibres alone, our subjects showed a small but significant elevation of tactile thresholds. This effect did not appear to be a result of changes in local skin mechanics given the absence of any change in tactile thresholds following the administration of a similar quantity of normal saline. However, it remains unclear whether the elevation of tactile thresholds following C-fibre blockade represents a direct role of C-LTMRs in normal tactile perception, or whether it represents an indirect role by way of a perturbation in the balance of peripheral inputs, neither of which precludes the role of this afferent class in tactile activity. Importantly, the loss of innocuous mechanoreception was only observed when T-type calcium channels were blocked in the compression condition, which, in conjunction in recent findings in mice [19] and convergent evidence from human pain work [20], suggests that the tactile sensitivity of C-LTMRs is regulated by Cav3.2 calcium channels.

C-LTMRs were first reported (in the cat hairy skin) in 1939 by Yngve Zotterman [5] but generated relatively little interest over the years, with a lack of evidence about their existence in humans, questions about their functional relevance to mechanoreception (in contrast to their myelinated counterparts), the pervasive notion that all cutaneous C fibres were nociceptors, and technical difficulties in identifying and recording from them likely to be amongst the deterring factors. After the initial discovery in the cat, it took many decades for C-LTMRs to be identified in non-human primates [6]. Likewise, it wasn’t until 1988 that this afferent class was discovered in the human (facial) skin [11] with a progressive corroboration of a more generalized distribution in the hairy skin areas in recent years, including both proximal (forearm/thigh) and distal (dorsal hand/lower leg) regions [13, 14, 23]. Indeed, whether C-LTMRs innervated the genitalia (across any type of species) was unclear until a recent study in mice, which, using TH to label C-LTMRs, found a sizable population of TH-positive neurons in the sacral DRG, hence making their presence in this region of skin quite likely [8]. It is plausible that similar limitations in the detection of C-LTMRs may extend to the glabrous skin that, once overcome, could reveal a glabrous representation. That notwithstanding, in a recent study the primary afferents expressing VGLUT3, which were anatomically and electrophysiologically defined as C-LTMRs, were shown to provide sensory innervation of mouse glabrous skin [17].

In the present study, the subjects found the quality of punctate touch following myelinated-fibre blockade to be nowhere near the complete/familiar sense of touch that was felt when all fibres were intact. This is consistent with findings in the two large-fibre deafferented patients (sensory neuronopathy syndrome, SNS) where “selective CT-stimulation [CT, C-tactile fibres] fails to evoke anything like a full sensation of pleasant touch” [24]. In contrast, the blockade of C fibres alone using intradermal anaesthesia had no discernible effect on the quality of touch. This may be because although it is posited that C-LTMRs mediate the affective/emotional attributes of touch such as the pleasantness associated with gentle brushing of the skin [25], the punctate mechanical stimuli used in the present study tend to be devoid of any overt affective element, at least in normal conditions. Another possibility is based on observations in cats, where Douglas and Ritchie [26] proposed that C-LTMRs may be more concerned with sub-perceptual processing. Indeed, this proposition has been reaffirmed with observations in the two SNS patients in whom gentle stroking of the skin resulted in infrequent reports of pleasantness that were plagued by marked inter- and intra-subject variances and only exceeded chance observation when forced-choice methods were used [24, 27]. This apparently weak contribution to perception is consistent with current findings that following compression subjects lost the sense of vibration; however, in the context of monofilament sensibility the effect was reproducible from trial to trial. It was important to test the tactile contribution of C-LTMRs in neurologically intact individuals in order to confirm that their proposed role in tactile activity in the two SNS patients was not merely a compensatory response to neuroplastic changes triggered by permanent loss of large myelinated afferents. In the current study, we attempted to circumvent the co-activation of myelinated fibres by employing nerve compression blocks. Following the block, all participants failed to detect vibration and cold, likewise the threshold for cold detection was reported well outside the thermal comfort zone, whereas warm/heat sensation remained intact. Under these conditions, low-force monofilament stimuli were detected by all participants across both skin types, and these stimuli were always perceived as non-painful. However, the possible role of other myelinated-fibre types that may still be conducting during a compression block (despite the absence of vibration and cold sensations) cannot be discounted. In saying that, the detectability of vibratory and thermal (cool/warm/heat-pain) stimuli was repeatedly tested during the compression block. In addition, the contribution of C-LTMRs to tactile activity was further tested by employing a low-dose anaesthetic block and T-type calcium channel antagonist. The findings of the present study, in addition to the observations in SNS patients [21] and the known responses properties of this afferent class [28], lend support to the contribution of C-LTMRs to normal tactile activity, in particular the detection of punctate tactile stimuli.

The somasthetic senses generally decline with normal aging, and these changes are often accentuated in pathological conditions such as peripheral neuropathies [29, 30]. Furthermore, in many neuropathic conditions, the onset of sensory changes coincides with selective or more pronounced abnormalities in small-fibre types relative to their large myelinated counterparts [31, 32]. In diabetic neuropathy for example, convergent evidence shows an elevation of tactile, thermal and pain thresholds (especially in distal limbs), a decrease in the absolute number of C-fibre endings in the skin, axonal atrophy (a reduction in axonal diameter, therefore reduced conduction velocity), and a characteristic shift in the relative proportion of C-fibre subtypes in favour of mechano-insensitive fibres, in addition to seemingly impaired capacity of their mechano-sensitive counterparts to transduce mechanical stimuli [31, 33–36]. Intriguingly, a severe loss of small myelinated and C fibres in some patients with advanced diabetic polyneuropathy was found to coincide with impairment/abolition of light touch sensation while the vibration sense, consistent with its large-fibre origin remained largely preserved [31]. Likewise, impaired mechanical detection with normal vibration detection was reported in the neuropathy associated with treated leprosy patients [37]. Although it is tempting to suggest that the loss of tactile sense could be due to a dysfunctional C-LTMR system, this remains a matter for conjecture. In saying that, it is noteworthy that the inhibition of Cav3.2 calcium channels resulted in abolition of allodynia in rats with painful peripheral diabetic neuropathy [38]. Regrettably, the role of small fibres in general remains less appreciated in part due to their ‘invisibility’ during routine nerve conduction testing [36, 39].

Based on nerve conduction block studies, much like this one, it is widely believed that the discriminative facet of tactile perception is underpinned exclusively by large myelinated afferents [1, 2]. Indeed, even in this study, vibration sense was lost during the compression block. However, a lack of percept need not preclude the activation of C-LTMRs, or any afferent class for that matter, to a given stimulus. Indeed, we have shown that the concurrent application of normally innocuous vibration to human hairy or glabrous skin during experimental cutaneous or muscle pain (or delayed-onset muscle soreness) could result in an augmentation of the overall pain intensity (allodynia). More importantly, this effect was found to persist whether or not the myelinated fibres were conducting [16, 22, 40, 41]. In these studies, the onset of allodynia tended to be delayed in the order of a few seconds, which indicated not just the conduction delay for C fibres but also the potential role of temporal summation in eliciting a perceptual response. Indeed, even in our current study, the blockade of myelinated fibres resulted in a small but conspicuous delay between the presentation of the stimulus and subsequent affirmation by the subject. Furthermore, a diminished appreciation of the onset and offset of skin indentation was reported. Moreover, the capacity to localise the site of tactile stimulation was seemingly impaired, much like the vague and diffuse expression of C-fibre mediated allodynia in ‘pain’ conditions. However, given the constraints of time during nerve conduction blocks, such observations could not be systematically tested.

There is no current evidence of a direct projection of C-LTMRs to the dorsal column nuclei (DCN), however, it has been suggested that the C fibres may gain access to the DCN through dorsal column postsynaptic fibres (arising from second-order dorsal horn neurons) that have been shown to respond to a range of noxious and innocuous stimuli including light touch [42–44]. At the dorsal horn level, the C-LTMR input is relayed to superficial laminae in a somatotopically organised manner [8]. Depending on the molecular/genetic markers used to label the spinal projection of C-LTMRs, they have been shown to terminate in lamina I [17], and the outer part [45] or the inner part [17] of lamina II or both regions [46]. In a recent electrophysiological study, it was reported that the lamina I neurons responsive to C-LTMRs are not specific for low-threshold tactile input, but can also be activated by noxious mechanical and heat stimuli, hence displaying the properties of ‘wide dynamic range’ neurons [47]. Large myelinated fibres have also been shown to terminate in the inner part of lamina II, in addition to lamina III/IV [48]. The functional significance of two distinct low-threshold tactile inputs to this region of dorsal horn, which has been implicated in injury-induced allodynia [48], remains unclear. Drew and MacDermott [49] conjectured that the dual innervation of the inner part of lamina II may allow for a C-LTMR driven amplification of large-fibre tactile input under injury conditions. Given the functional peculiarity of this dorsal horn region, it might be inferred that such a mechanism could underpin a modality crossover following injury (e.g. tactile allodynia), in addition to preserving a crude tactile map providing spatial cues for the overall experience.

## Conclusions

The data obtained from the compression experiments serve as further evidence for the presence for an underlying network of C-low threshold mechanoreceptors in human glabrous skin that, much like their hairy skin counterparts, can encode mechanical punctate stimuli. Furthermore, the data obtained from the low-dose anaesthesia and T-channel antagonist experiments suggest that the Cav3.2-expressing low-threshold C afferents are not necessarily a redundant tactile system, but rather they appear to contribute to normal tactile detection, although whether this is in a direct or facilitatory role is not known. Indeed, behavioural models of neuropathic pain typically rely on withdrawal responses to punctate tactile stimulation of glabrous skin areas as a measure of injury-induced ‘nociceptive’ activity. In light of the recently discovered role of C-LTMRs in allodynia, and the findings of the current study in conjunction with reports from SNS patients, further examination of the contribution of C-LTMRs to touch and pain processing is warranted.
